# Clinical characteristics of Japanese patients with axial spondyloarthritis, and short-term efficacy of adalimumab

**DOI:** 10.1007/s00776-015-0755-z

**Published:** 2015-08-06

**Authors:** Akiyo Otsuka, Mitsuhiro Morita, Harumoto Yamada

**Affiliations:** Department of Orthopaedic Surgery, Fujita Health University, 1-98, Dengakugakubo, Kutsukake-cho, Toyoake, Aichi 470-1192 Japan

## Abstract

**Background:**

Ankylosing spondylitis (AS) is rarer in Japan than in Europe, probably because the European criteria, not well known by Japanese general physicians, regard AS as a progressive stage of axial spondyloarthritis (SpA). HLA-B27 is an important diagnostic marker of SpA; however, the incidence of the HLA-B27 allele is as low as 0.4 % in Japan. For Japanese SpA patients, other HLA alleles and clinical findings are required for earlier definitive diagnosis, for determining appropriate treatment timing, and for disease monitoring.

**Methods:**

We investigated the HLA-B alleles of 36 patients clinically diagnosed with SpA. For 8 axial SpA patients we evaluated the short-term efficacy of subcutaneous adalimumab injections (40 mg every other week for ≥11 months). Treatment efficacy was evaluated by use of the Bath Ankylosing Spondylitis Activity Index (BASDAI) score, and serum TNF-α and IL-6 levels were measured pre and post-treatment.

**Results:**

Among the 36 Japanese SpA patients, the HLA-B27 allele occurred infrequently (5.6 %) whereas the HLA-B44 and 61 alleles were the most frequently detected (25.0 %). We also detected severe bamboo spine on radiography in the absence of the HLA-B27 allele. All 8 patients with axial SpA experienced significant symptom improvement after adalimumab treatment; the HLA-B27 allele was absent from these patients. Serum TNF-α and IL-6 levels were elevated in cases with remarkable inflammatory pain and high disease activity. These cytokines decreased after therapy, however. Most patients with normal cytokine levels at baseline retained these low levels.

**Conclusions:**

The findings reveal the short-term efficacy of adalimumab. The remarkably low incidence of HLA-B27 among our patients indicates that HLA-B distribution is different from that in other countries. Serum TNF-α and IL-6 levels were not effective as biomarkers for cases without high disease activity, and further research with larger samples is needed. The efficacy of TNF blockers, however, suggested a potential localized TNF effect was present among SpA patients.

## Introduction

Spondyloarthritis (SpA) is a group of several related but phenotypically distinct disorders that are categorized into axial SpA and peripheral SpA, on the basis of primary symptom location. According to the most recent Assessment of Spondyloarthritis international Society (ASAS) definition, predominantly axial SpA includes the categories non-radiographic, radiographic axial SpA, and ankylosing spondylitis (AS), which is regarded as the most well-known SpA type, and predominantly peripheral SpA, which includes psoriatic arthritis, reactive arthritis, enteropathic arthritis, and undifferentiated SpA. Other SpA types include acute anterior uveitis, juvenile SpA, and SAPHO (synovitis, acne, pustulosis, hyperostosis, and osteitis). The primary symptom of axial SpA is insidious inflammatory back pain, which is often misdiagnosed because its clinical characteristics are not widely recognized by orthopedic doctors. Diagnosis is, therefore, often delayed by several years [1].

SpA is rarer in Japan than among Caucasians. The incidence is 0.48/100,000 and the prevalence 9.5/100,000 person-years among the Japanese, less than 1/10 and 1/200, respectively, of those among Caucasians [2]. The relatively low values may be the result of the misdiagnosis of SpA as mechanical back pain by orthopedic doctors in Japan. Shichikawa et al. [3] reported similar prevalence of SpA and rheumatoid arthritis (RA) in Japan.

Several criteria are available for diagnosis of SpA. The earliest were the modified New York criteria for AS (1984). The Amor criteria for SpA (1990), the ASAS criteria for classification of axial SpA (2009), and the ASAS criteria for classification of peripheral SpA (2011) depend on the presence of HLA-B27. A decision tree for axial SpA diagnosis, presented by Rudwaleit et al. [4], enables diagnosis of early-stage AS, or non-radiographic SpA, which cannot be categorized by use of the modified New York criteria because it requires radiological changes in the sacroiliac joint for diagnosis. Similar to the other criteria, HLA-B27 is an important part of the decision tree; however, inclusion of HLA-B27 makes it challenging to diagnose SpA among Japanese patients because of the low incidence of HLA-B27, reportedly as low as 0.4 % [5].

Traditional treatment options for SpA in Japan include non-steroidal anti-inflammatory drugs (NSAIDs), systemic corticosteroid therapy, and disease-modifying antirheumatic drugs (DMARDs). Since 2010, adalimumab, a recombinant fully human anti-tumor necrosis factor (TNF) monoclonal antibody, has been available for treatment of SpA patients for whom the disease if poorly controlled by use of traditional therapy. The efficacy of adalimumab has been reported in several countries [6–8]. Recent studies have shown that early treatment with TNF-blockers can achieve a higher clinical response for AS and nonradiographic SpA [9, 10].

Therefore, appropriate criteria and biomarkers to aid in diagnosis are needed to enable early use of anti-TNF therapy among Japanese SpA patients.

Owing to the low incidence of HLA-B27 in Japan and the presence of increased TNF concentrations in the joints of patients with AS [11, 12], serum TNF-α levels could better reflect SpA disease activity. Here, we evaluated serum cytokines as effective biomarkers of SpA, to assist with early diagnosis and assessment of adalimumab treatment efficacy. Because some of our patients with typical radiographic SpA, for example bamboo spine, were HLA-B27 negative, we also investigated the variations in HLA-B alleles and clinical characteristics of Japanese SpA patients for early definitive diagnosis. The purpose of this study was to evaluate the efficacy of adalimumab and to investigate the variation of HLA-B alleles among Japanese SpA patients. We also evaluated serum cytokines as effective biomarkers of SpA diagnosis and disease monitoring.

## Methods

### Patients

We investigated HLA-B alleles among 36 patients who were clinically diagnosed with SpA in our institutes and who agreed to pay for examination of the HLA alleles. Eight of the 36 patients were treated with adalimumab. All 8 patients experienced enthesitis or insidious inflammatory pain in the lower back or sacroiliac joints; they also met the definition of AS based on the modified New York criteria. All of the patients provided informed consent.

The study was approved by the local ethics committee of Fujita Health University and conducted in accordance with the World Medical Association Declaration of Helsinki.

Despite previous treatment, which had included one or more NSAIDs, opioids, or systemic corticosteroid therapy, 5 of the 8 patients had active disease, as defined by use of the total Bath Ankylosing Spondylitis Activity Index (BASDAI) score on a visual analog scale (VAS) ≥4 cm, for >4 weeks at the time of adalimumab initiation, and the remaining 3 patients had experienced insidious back pain for more than 6 months. All 8 patients were administered adalimumab 40 mg subcutaneously every other week for at least 11 months.

### Data collected

Patient background was investigated by use of the HLA-B allele, serum cytokine concentrations of TNF-α and IL-6 (LSI Medience, Tokyo, Japan), disease duration, and radiographic examination of the sacroiliac joint and the whole spinal column.

### Outcome evaluations

The total BASDAI and specific item scores were used to evaluate the subjective outcomes at baseline and at every medical consultation after initiation; the specific item scores included fatigue, spinal pain, peripheral arthritis, localized tenderness (enthesitis), intensity of morning spinal stiffness, and duration of morning stiffness. The objective outcomes were evaluated by use of biological examinations: serum levels of C-reactive protein (CRP), macrophage-derived cytokines (tumor necrosis factor [TNF]-α and interleukin [IL]-6), and erythrocyte sedimentation rate (ESR) (LSI Medience, Tokyo, Japan). CRP levels and ESRs were measured at every medical examination, and TNF-α and IL-6 levels were measured at baseline and the time of the last observation.

The average and standard deviation of the BASDAI values for each of the 6 items were measured and compared by use of paired *t* tests; *p* < 0.05 was regarded as indicative of a significant difference.

## Results

The age of the 36 Japanese patients (13 men, 23 women) who underwent the HLA test was 23–87 years. The average age of the 8 Japanese patients (2 men, 6 women) treated with adalimumab was 55.8 years, and mean axial SpA disease duration was 20.9 years (Table 1).

**Table 1:** Characteristics of patients with spondyloarthritis

Case	Age/sex	DD (years)	HLA-B	X-ray grade^a^	Remarkable findings and past history	Enthesitis	Uveitis	IBD	Concomitant medication
1	52/M	20	B7, B61	IV, IV	Bamboo spine	−	−	−	NSAID
2	53/M	35	B46, B52	IV, IV	Syndesmophyte, peripheral type	+	−	−	Methotrexate, NSAID
3	27/F	10	B44, B35	II, III	Iliac joint sclerosis	−	−	−	−
4	76/F	5	B13, B52	II, II	Total hip arthroplasty	−	−	−	−
5	51/F	30	B44, B59	II, III	Enthesitis	+	−	−	Methotrexate, tramadol, acetaminophen
6	63/F	10	B46, B51	II, II	Vertebral sclerosis, SAPHO	−	−	−	−
7	78/F	40	B44, B35	II, II	Aortic valve insufficiency	−	−	−	−
8	46/F	17	B62, B35	II, II	Axial and peripheral type	−	−	−	Tramadol, acetaminophen

*DD* disease duration, *IBD* inflammatory bowel disease, *NSAID* non-steroidal anti-inflammatory drug, *SAPHO* synovitis, acne, pustulosis, hyperostosis, osteitis

^a^ X-ray grade of the sacroiliac joint

On radiography, one of the male patients had severe changes for example a bamboo spine, whereas the other male patient had severe enthesitis. For 2 of the patients the radiological grade of the bilateral sacroiliac joints was IV. Case 2 developed both peripheral symptoms and enthesitis, and Case 8 had peripheral symptoms. Methotrexate (MTX) was added to the adalimumab therapy for 2 patients with enthesitis (Cases 2 and 5). Case 4 underwent total hip arthroplasty for osteoarthritis. Case 6 had previously been diagnosed with SAPHO syndrome at another hospital, and vertebral sclerosis was observed on radiology. Case 7 underwent a cardiac operation for aortic valve insufficiency. None of the 8 patients developed uveitis or inflammatory bowel disease (Table 1). No patient was positive for the HLA-B27 allele. Of the 9 HLA-B allele types (B7, B27, B35, B39, B44, B51, B52, B61, and B62) that are reportedly typical of Japanese SpA patients [13], B35 and B44 were found for 3 patients, B52 was found for 2 patients, and B7, B51, B61, and B62 were each found for one patient (Table 2).

**Table 2:** Gene frequencies of HLA-B alleles in patients with spondyloarthritis

HLA-B	Number of cases (Case #)	Frequency (%)
B7	1 (1)	12.5
B27	0	0
B35	3 (3, 7, 8)	37.5
B39	0	0
B44	3 (3, 5, 7)	37.5
B51	1 (6)	12.5
B52	2 (2, 4)	25.0
B61	1 (1)	12.5
B62	1 (8)	12.5

Of the 36 Japanese patients, radiographic axial SpA was observed for 13, and 2 of these 13 were positive for the HLA-B27 allele. Non-radiographic axial SpA was observed for 23 of the 36 patients; all of the 23 were negative for the HLA-B27 allele. The B44 and B61 alleles were the most frequently found (25.0 %); the B35 (22.2 %) and B51(19.4 %) alleles were also frequently found (Table 3).

**Table 3:** Gene frequencies of HLA-B alleles among patients with spondyloarthritis in our study compared with those reported in previous Japanese studies [12, 30]

HLA-B	Our patient population (*n* = 36) (%)	Our cases (*n* = 8) (%)	Previous reports from Japan (%)	Healthy population (%)
B7	11.1	12.5	11.5	5.2
B27	5.6	0	0.4	0.3
B35	22.2	37.5	13.8	8.6
B39	0	0	10.7	4.4
B44	25.0	37.5	13.0	7.9
B51	19.4	12.5	15.0	8.0
B52	16.7	25.0	24.5	13.9
B61	25.0	12.5	23.7	14.6
B62	8.3	12.5	14.2	7.2

**Fig. 1:**
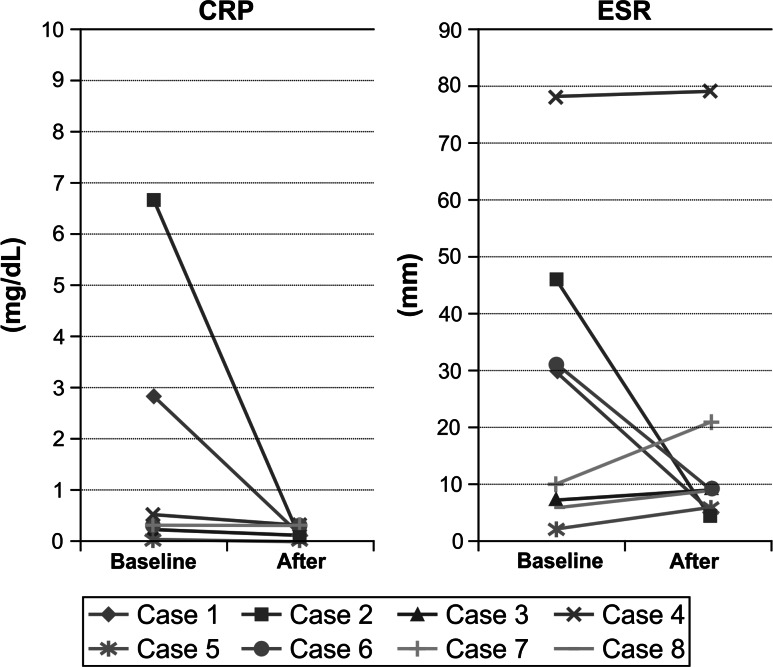
C-reactive protein (CRP) levels and erythrocyte sedimentation rates (ESRs) among patients with spondyloarthritis, before and after treatment with adalimumab

CRP levels were elevated at baseline for 3 of the 8 patients undergoing adalimumab therapy, and all became normal at the last examination. For 5 patients, CRP levels remained normal during the therapy. ESRs were elevated for 4 patients at baseline; after therapy they returned to normal for 3 patients and remained the same for 1 patient. For the 4 patients for whom ESR was normal, at the latest observation it remained normal for 3 patients and reached an elevated level for 1 patient (Fig. 1).

**Fig. 2:**
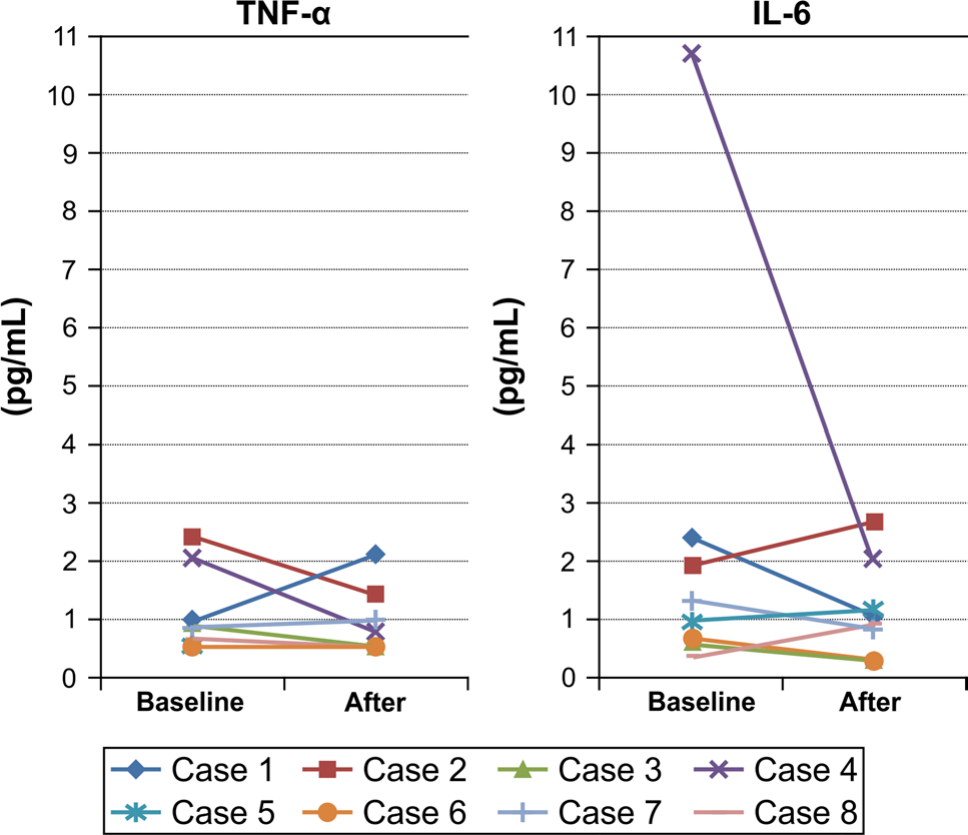
Serum tumor necrosis factor (TNF)-α and interleukin (IL)-6 levels among patients with spondyloarthritis, before and after treatment with adalimumab

Serum TNF-α levels were abnormal (≥1.79 pg/dL) for 2 patients at baseline and became normal at the last observation for both patients. One patient with a normal TNF-α level at baseline had an abnormal level at the last observation. The levels for 5 patients remained normal: decreased for 2 patients, increased for 1 patient, and remained under the threshold for 2 patients (0.55 pg/mL) (Fig. 2).

The serum IL-6 level was elevated (≥2.56; 10.7 pg/mL) for 1 patient at baseline and reached normal levels (2.01 pg/dL) at the last observation. One patient who had a normal baseline serum IL-6 level had an elevated level at the last observation, and 6 patients had normal serum IL-6 levels (that decreased for 4 and increased for 2 patients) (Fig. 2).

**Fig. 3:**
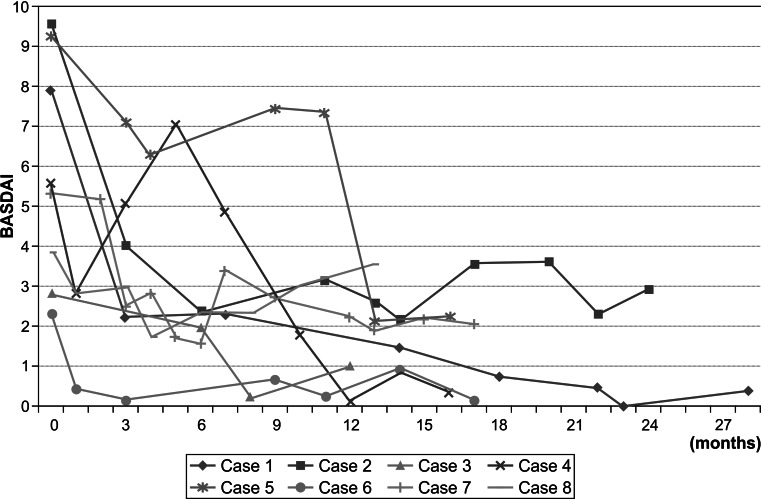
Changes in the Bath Ankylosing Spondylitis Activity Index (BASDAI) scores for patients with spondyloarthritis after treatment with adalimumab

The total BASDAI score improved for all patients compared with the baseline score (Fig. 3). Seven (87.5 %) patients achieved 50 % reduction in the initial BASDAI score (BASDAI50 response = remarkably effective). Four (50 %) patients experienced rapid clinical improvement and achieved a BASDAI50 response within 3 months after adalimumab initiation. One (12.5 %) patient achieved BASDAI50 between 3 and 6 months, and 2 (25 %) patients achieved BASDAI50 within 6–12 months.

One patient became drug-free; another patient who had secondary failure dropped out of the study at 11 months. We observed no adverse events, including local injection fraction, among these cases during adalimumab therapy.

**Fig. 4:**
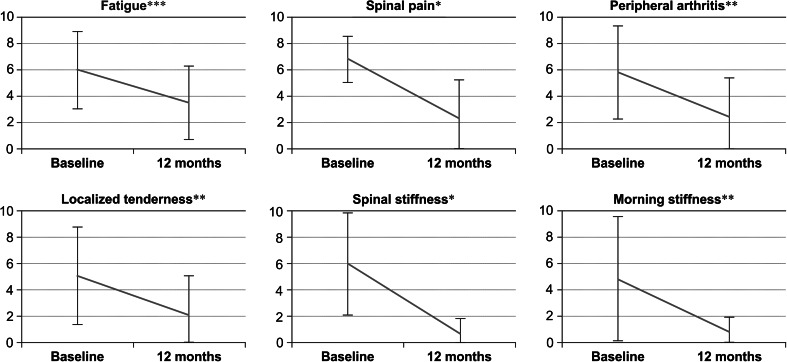
Changes in each of the mean Bath Ankylosing Spondylitis Activity Index (BASDAI) item scores for patients with spondyloarthritis, before and after treatment with adalimumab *p < 0.01, **p < 0.05, ***p < 0.1

Figure 4 illustrates the mean scores of each of the 6 BASDAI items at baseline and at the last examination. All 6 items improved after 12 months of adalimumab therapy, with significant alleviation of back pain (*p* < 0.01), the intensity of morning spinal stiffness (*p* < 0.01), peripheral arthritis (*p* < 0.01), and the duration of morning stiffness (*p* < 0.05).

### Cases

**Fig. 5:**
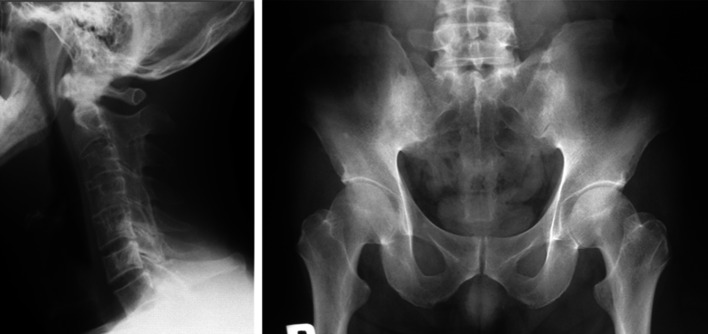
Radiography of the bamboo cervical spine (*left*) and bilateral sacroiliac joints (*right*) of case 1

*Case 1.* A 52-year-old man with bamboo spine had been experiencing middle back pain and neck stiffness for more than 20 years. The patient was HLA-B7 and B61 positive. His cervical spine had the typical characteristics of bamboo spine (Fig. 5 left), and a syndesmophyte was present in the lumbar spine. The Modified New York radiological sacroiliitis criteria were grade 4 (bilateral) (Fig. 5 right). Serum CRP, ESR, and IL-6 levels were elevated before adalimumab therapy, and improved to normal levels after the therapy. The BASDAI score improved remarkably, from 7.9 to 2.2 after the first injection.

**Fig. 6:**
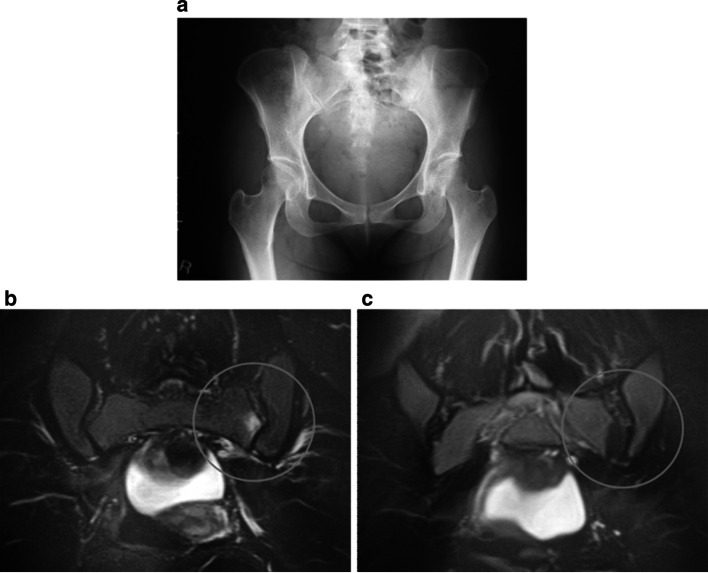
Radiography of the sacroiliac joints (**a**) and STIR sequence MRI for 1 patient with spondyloarthritis before (**b**) and after (**c**) treatment with adalimumab

*Case 2.* A 27-year-old woman with non-radiographic axial SpA had clinically suspected axial SpA for 10 years. The patient was HLA-B27 negative and B44 and B35 positive. The Modified New York radiological sacroiliitis criteria were grades 2 (right) and 3 (left) (Fig. 6a). The area of the hyperintense signal from the sacroiliac joint on short time inversion recovery (STIR) sequence magnetic resonance imaging (MRI) reflected bone marrow edema, which is the active inflammatory phase of sacroiliitis (Fig. 6b). After 12 months of adalimumab therapy, the area of hyperintense signal was of lower intensity (Fig. 6c). The patient’s low back pain had also resolved.

## Discussion

The results of this study indicate that adalimumab was effective for short-term management of the symptoms of SpA which was not apparent from serum TNF-α or IL-6 levels. In addition, the incidence of HLA-B27 in this sample was remarkably low.

For Western and Japanese patients, TNF inhibition therapy is highly effective for alleviating the signs and symptoms of AS and non-radiographic axial SpA [6–8, 14–17]. With adalimumab therapy for non-radiographic SpA patients from America, Europe, and Australia, the mean BASDAI change was −1.9 at the primary endpoint at 12 weeks [6]. The previous study of adalimumab therapy among Japanese patients with AS achieved −3.4 (−4.2 to −2.7) at week 12 [8]. Similarly, this study achieved −2.5 (−5.7 to −0.4) change of the mean BASDAI at week 12. Haibel reported a greater clinical response to TNF-blockers with earlier treatment of AS and nonradiographic axial SpA [9, 10] Furthermore, compliance with the adalimumab therapy probably contributed to the observed, rapid clinical improvement in this study.

Previous studies have revealed increased macrophage-derived cytokine (TNF-α and IL-6) levels among patients with AS, and a relationship between IL-6 and disease activity has been reported [11, 12, 18–20]. In this present study, serum TNF-α levels remained the same after adalimumab administration. Serum IL-6 levels significantly decreased after adalimumab administration for 1 (12.5 %) patient only, whereas levels remained normal for 5 (62.5 %) patients. Therefore, even after the significant efficacy of cytokine therapy (TNF blockers and anti-IL-6 receptor antibody [21]), other, yet unknown, factors may explain the pain and disease activity in SpA. Until we have a better understanding of the underlying mechanisms, we suggest the use of serum TNF-α and IL-6 levels to determine the appropriate timing of treatment and to monitor disease activity among patients with greater disease activity and abnormally high cytokine levels. For patients with less disease activity and normal cytokine levels, the importance of cytokine levels as biomarkers remains to be determined. Individual differences may exist in the usefulness of serum TNF-α and IL-6 levels as biomarkers for diagnosis and evaluation of disease activity.

Previous reports have indicated that traditional DMARDs, for example MTX and sulfasalazine (SASP), are not effective for treatment of AS and axial SpA [22, 23] whereas SASP may be effective for peripheral SpA [24, 25]. Two of the patients in this study with severe enthesis and peripheral symptoms experienced greater alleviation of their peripheral symptoms on addition of MTX to their adalimumab therapy. Therefore, the combined use of MTX with adalimumab may be effective for enthesis and peripheral symptoms of SpA, and the efficacy may depend on each case.

SpA is believed to be strongly associated with HLA-B27, the incidence of which is reportedly low (0.4 %) in Japan. The frequency of HLA-B27 [26] and distribution of HLA-B differ, depending on ethnicity, with reported differences among SpA patients in Mexico [27], Tunisia [28], Alaskan Eskimos [29], and Sub-Saharan Africa [30]. Among 253 Japanese SpA patients, the frequency of HLA-B27 was low whereas the frequencies of B7, B35, B39, B44, B51, B52, B61, and B62 were high [13]; this is similar to the findings in this study (Table 3).

HLA-B27 as a diagnostic criterion for SpA may, therefore, not have much importance for Japanese patients. Instead, HLA-B typing should be considered for Japanese SpA patients, particularly as dependence on HLA-B27 can delay SpA diagnosis for Japanese patients.

We suspect that the clinical presentation of SpA (including AS) is also quite different for Japanese patients from that for European patients. Japanese SpA patients who meet the ASAS criteria are more likely to be women, even though more men are expected to meet the diagnostic criteria. Fibromyalgia (FM) symptoms are similar to those among Japanese female SpA patients, and we found it difficult to discriminate between nonradiographic axial SpA and FM in a few cases. In addition, progression to more severe symptoms, for example bamboo spine, in AS may not occur as frequently among Japanese SpA patients as among European patients. Additional studies are needed to enable understanding of the characteristics of Japanese patients.

The findings of this study reveal the short-term efficacy of adalimumab for 8 Japanese SpA patients. The remarkably low incidence of HLA-B27 indicates that variations in HLA-B types and symptoms among Japanese SpA patients are different from those among SpA patients of other countries. Serum TNF-α and IL-6 levels were not effective as biomarkers for cases without high disease activity, and further research with larger samples is needed.

A limitation of this study is that not all patients with clinically suspected SpA provided consent for the HLA test because the fee is not covered by health insurance, and it is very expensive (approximately 30,000 yen, or ~$250) for typical patients in Japan. Second, the numbers of samples for serum cytokines were not large enough for determination of their effectiveness as biomarkers.

## Conclusion

The findings of our study reveal the short-term efficacy and usefulness of adalimumab. The remarkably low incidence of HLA-B27 indicates that HLA-B distribution is different from that among SpA patients in other countries. Serum TNF-α and IL-6 levels were not effective as biomarkers in the cases without high disease activity, and further research with larger samples is needed.
